# Advances in pelvic imaging parameters predicting surgical difficulty in rectal cancer

**DOI:** 10.1186/s12957-023-02933-x

**Published:** 2023-02-27

**Authors:** Qingbai Zhang, Jiufeng Wei, Hongsheng Chen

**Affiliations:** Department of General Surgery, The Fourth Affiliated Hospital of Harbin Medical University, Harbin, China

**Keywords:** Rectal cancer, Difficult pelvis, Laparoscopic surgery, Difficulty of surgery, Preoperative prediction

## Abstract

Due to the fixed bony structure of the pelvis, the pelvic operation space is limited, complicating the surgical operation of rectal cancer, especially middle and low rectal cancer. The closer the tumor is to the anal verge, the smaller the operative field and operating space, the longer the operative time, and the greater the incidence of intraoperative side injuries and postoperative complications. To date, there is still no clear definition of a difficult pelvis that affects the surgical operation of rectal cancer. Few related research reports exist in the literature, and views on this aspect are not the same between countries. Therefore, it is particularly important to predict the difficulty of rectal cancer surgery in a certain way before surgery and to select the surgical method most suitable for each case during the treatment of rectal cancer.

## Introduction

The latest statistics show that the global incidence of colorectal cancer ranks third among malignant tumors, while its mortality rate ranks second [1]. In China, the incidence rate of rectal cancer is much higher than that in developed countries, and it shows a younger trend [2]. Since Heald et al. first proposed the idea of total mesorectal excision (TME) in 1982 [3], TME has become the gold standard for surgical treatment of mid–low rectal cancer. The difficulty of TME lies in the need for the operator to perform a complete resection of the lesioned rectum and its surrounding mesentery in the pelvis. However, due to the fixed bony structure of the pelvis, the space available for pelvic operation is limited, which can complicate the procedure, especially when treating middle and low rectal cancer. The study by Vaccaro et al. confirmed that the conversion rate of laparoscopic rectal cancer surgery to laparotomy was significantly higher than that of colon surgery [4]. The rectum is located in the small funnel-shaped pelvis. The closer the tumor is to the anal verge, the smaller the surgical field and the operating space. It is particularly difficult to dissociate, cut-off, and anastomose the distal rectum, making the operation more complex, and the operation time can vary significantly [5]. In the case of anatomical dissection and TME in the low pelvis, the possibility of a surgical side injury, such as an intraoperative ureteral injury, presacral hemorrhage, postoperative urinary dysfunction, sexual dysfunction, or anastomotic leakage, will increase [6]. In addition, the narrow operating space can lead to incomplete resection specimens—even a positive circumferential resection margin—or other adverse consequences [7]. In recent years, with the development of minimally invasive techniques, emerging technologies, such as transanal total mesorectal excision (taTME) and robotic surgery, have provided more options for the treatment of rectal cancer. taTME combines a bottom-up surgical strategy with the concept of TME, and it may be easier to perform low rectal dissection and mesenteric resection in the deep and narrow pelvis using this approach [8, 9], providing the possibility of sphincter preservation for low rectal cancer. In addition, robotic surgery can overcome the difficulties brought on by the pelvic anatomy, in that the robot has multiple robotic arms that can rotate 360° and can perform operations that cannot be completed by human hands or using conventional laparoscopic instruments. Distinguishing anatomical levels is easier with this approach, yet these robots are expensive. At present, considering the additional economic and time overhead, robotic surgery may only be selectively applied to those patients who would benefit from this new technology.

To sum up, rectal cancer has a long operation time, a high complication rate, and many surgical methods. Therefore, it is particularly important to predict the difficulty of rectal cancer surgery before the operation and to select the best surgical method suitable for individual patients. There is still no clear definition of a difficult pelvis that affects the operation, with few related research reports available in the literature, and the views on this aspect are not the same between countries. This article presents a systematic review of the relative imaging parameters measured by the pelvis in recent years that influence the difficulty of surgery, the positive rate of circumferential resection margin, the integrity of surgical specimens, and the prognosis of rectal cancer.

## Imaging parameters

It is particularly important to establish an effective evaluation system for the difficulty of rectal cancer surgery according to the operable space of the pelvis. In the past, pelvic measurement was mostly performed in obstetrics and gynecology; however, with the continuous exploration of laparoscopic technology, in recent years, many colorectal experts at home and abroad have also turned their attention to pelvic measurement. The pelvis is a complete bony ring formed by connections between the left and right hip bones, the sacrum, and the coccyx. The pelvis may be divided into two parts by an oblique line (from the back of the sacral promontory, through the iliac arcuate line, the idiopubic eminence, the pubic comb, the pubic tubercle, the pubic tubercle, and the line connecting the pubic crest to the upper border of the symphysis pubis); the area above this line is referred to as the false pelvis, and that below it is known as the true pelvis. The true pelvis, also known as the small pelvis, has an uneven diamond-shaped outlet formed by the tip of the coccyx and the sacrotuberous ligaments on both sides, the ischial tubercle, the pubic arch, and the lower border of the pubic symphysis [10, 11].

The pelvic measurement methods that can be used in the treatment of rectal cancer in the clinic are mainly magnetic resonance imaging (MRI) and computed tomography (CT). The advantages of CT pelvic measurement are its low radiation dose, rapid and accurate scanning, and comfortable examination process. Two-dimensional images of CT pelvic measurements and corresponding anatomical diameters have been used in many studies; however, the pelvic parameters of two-dimensional images are only images of various cross-sections, which cannot express the shape of the pelvis three-dimensionally (3D), while thin-slice spiral CT imaging can reconstruct the pelvis in 3D [12] so that the operator can measure the pelvis on any axis, allowing for its more accurate assessment. However, 3D image reconstruction requires expensive software, sophisticated technology, and skilled personnel who can operate the relevant systems. Therefore, 3D CT pelvic reconstruction is not a routine process for patients with rectal cancer. Compared to CT, non-radioactive MRI has many advantages for the preoperative evaluation of rectal cancer [13]: not only does it have the advantage of high accuracy in measuring pelvic anatomy but also those of reconstructing pelvic floor muscles, nerves, and blood vessels, and it can display the soft tissue around the rectum more clearly and can more accurately detect the state of the mesorectal fascia and its submicroscopic lymph nodes. The depth of tumor invasion into the mucosa and suspicious lymph node metastasis in the perirectal mesentery can also be assessed by MRI [14]. At present, some pelvic anatomical parameters to assess the difficulty of surgery are based on preoperative MRI pelvic measurements [14–16]. In the future, preoperative MRI may not only be used for accurate measurement of the pelvis but also to simulate surgery and other fields, which would have high clinical value in rectal cancer–related applications [17, 18].

Table 1 is an overview of the related positive imaging parameters based on CT/MRI that have been used more frequently to reduce the difficulty of rectal cancer surgery in recent years. As can be seen from Table 1, the small pelvis parameters such as interspinous diameter of the ischial spine, anteroposterior diameter of the pelvic outlet, and mesorectal fat area have received more research attention. The size of pelvic parameters and the degree of mesorectal fat area appear to be better predictors of the difficulty of rectal cancer surgery.

**Table 1:** Imaging parameters

Pelvic imaging parameters	Description (see illustration for details)	References
(a) Anteroposterior diameter of the pelvic entrance	The line connecting the midline of the superior border of the symphysis pubis to the sacral promontory (Fig. 1)	[6, 19–21]
(b) Pelvic entrance transverse diameter	The maximum distance between the iliopubic lines on both sides (Fig. 2)	[6, 20]
(c) Anteroposterior diameter of the middle pelvis	The line connecting the midpoint of the inferior border of the pubic symphysis to the middle of the anterior border of the sacrococcygeal junction (Fig. 1)	[19, 22]
(d) Anteroposterior diameter of the pelvic outlet	The line connecting the midpoint of the inferior border of the pubic symphysis to the tip of the coccyx (Fig. 1)	[19, 20, 23]
(e) Pelvic outlet diameter	The line between the sciatic spines on both sides (Fig. 3)	[6, 15, 20, 22–29]
(f) Pubic symphysis height	The line in the middle of the upper and lower edges of pubic of symphsis (Fig. 1)	[19, 22, 29]
(g) Sacrococcygeal distance	The line connecting the midline of the anterior border of the sacral promontory to the tip of the coccyx (Fig. 1)	[19, 23, 29, 30]
(h) Sacrococcygeal curvature depth	A vertical line from the deepest part of the sacrococcygeal hollow to the sacrococcygeal distance (Fig. 1)	[19]
(i) Sacrococcygeal–pubic angle	The angle between the extension line of the anteroposterior diameter of the pelvic inlet and the extension line of the anteroposterior diameter of the pelvic outlet (Fig. 1)	[19]
(j) Pelvic depth	The line connecting the midpoint of the anteroposterior diameter of the pelvic entrance to the tip of the coccyx (Fig. 1)	[7, 19]
(k) Mesorectal fat area	The area of mesangium and fat around the rectum at the level of the tip of the sciatic spine (Fig. 3)	[13, 23, 25, 26]
(l) Angle T4	The angle between the upper and lower borders of the pubic symphysis with the lower border of the tumor as the vertex (Fig. 4)	[31]
(m) Angle A5	The angle between the line connecting the upper and lower borders of the pubic symphysis and the line connecting the midpoint of the upper border of the pubic symphysis to the sacral promontory (Fig. 1)	[16]

**Fig. 1:**
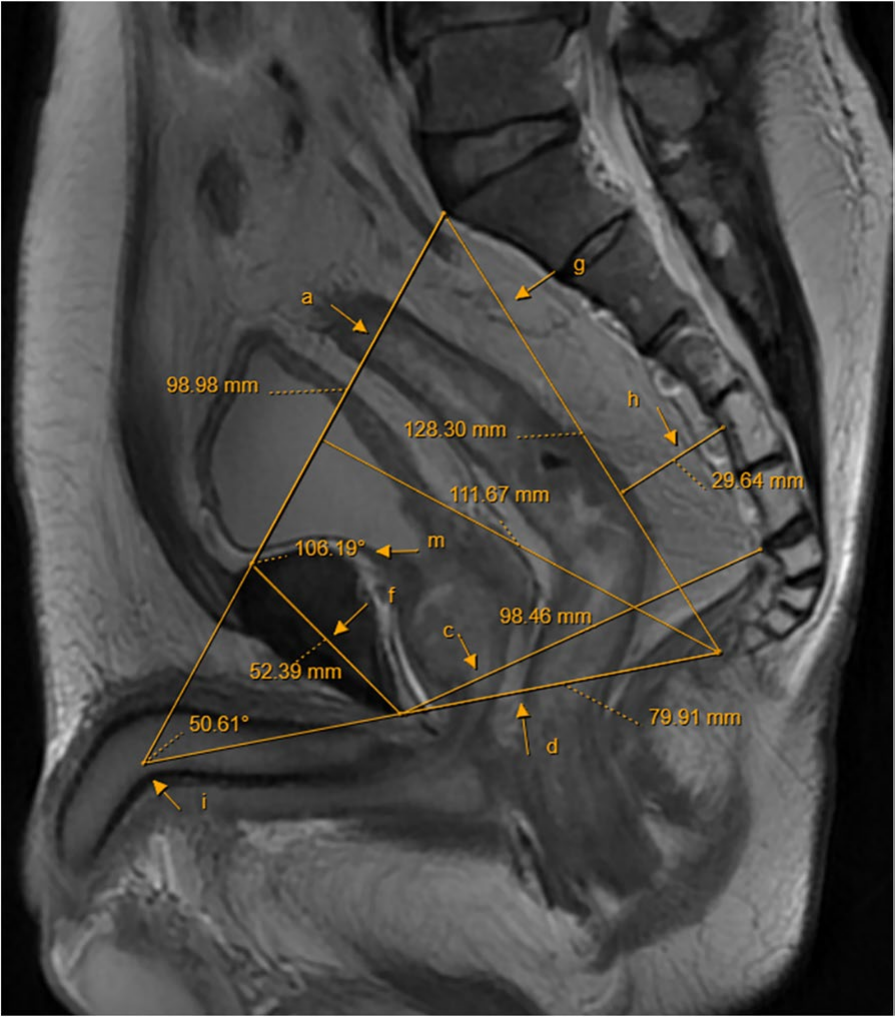
Sagittal pelvic magnetic resonance images: **a** anteroposterior diameter of the pelvic entrance, **c** anteroposterior diameter of the middle pelvis, **d** anteroposterior diameter of the pelvic outlet, **f** pubic symphysis height, **g** sacrococcygeal distance, **h** sacrococcygeal curvature depth, **i** sacrococcygeal–pubic angle, **j** pelvic depth, and **m** Angle A5

**Fig. 2:**
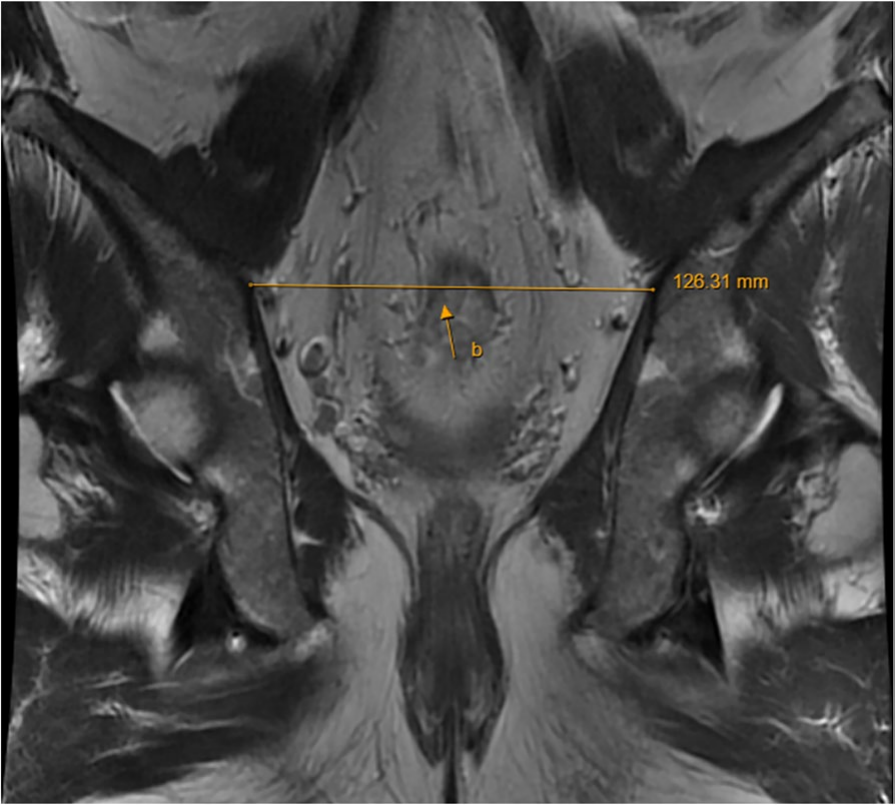
Coronal MRI of the pelvis: **b** pelvic entrance transverse diameter

**Fig. 3:**
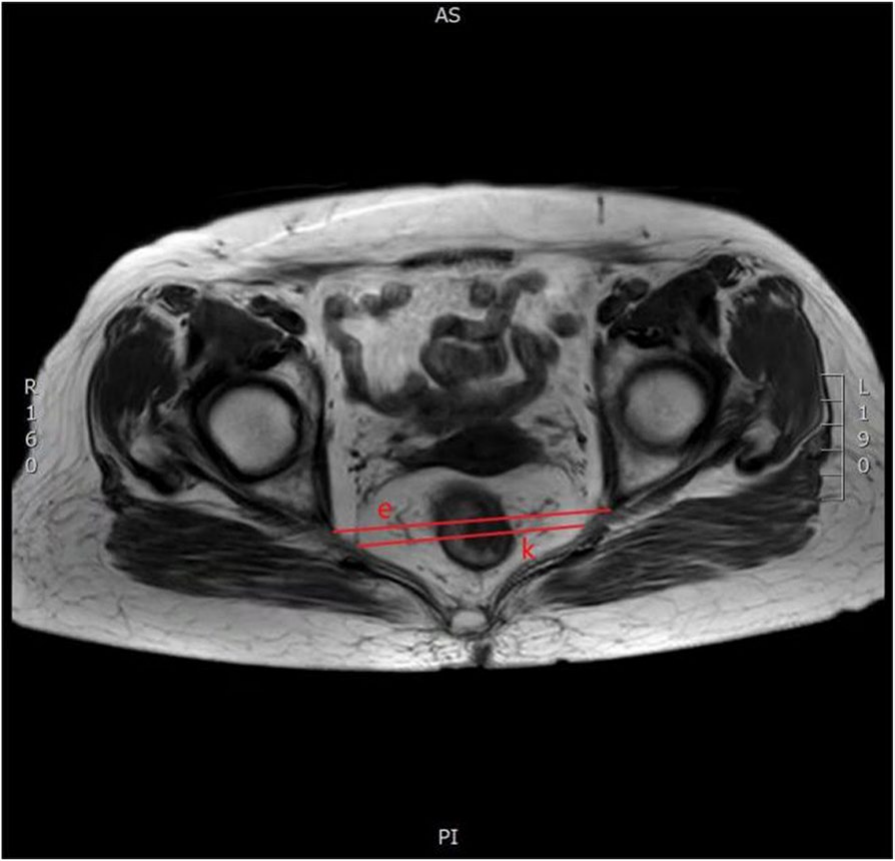
Horizontal pelvic magnetic resonance images: **e** pelvic outlet transverse diameter and **k** mesorectal fat area

**Fig. 4:**
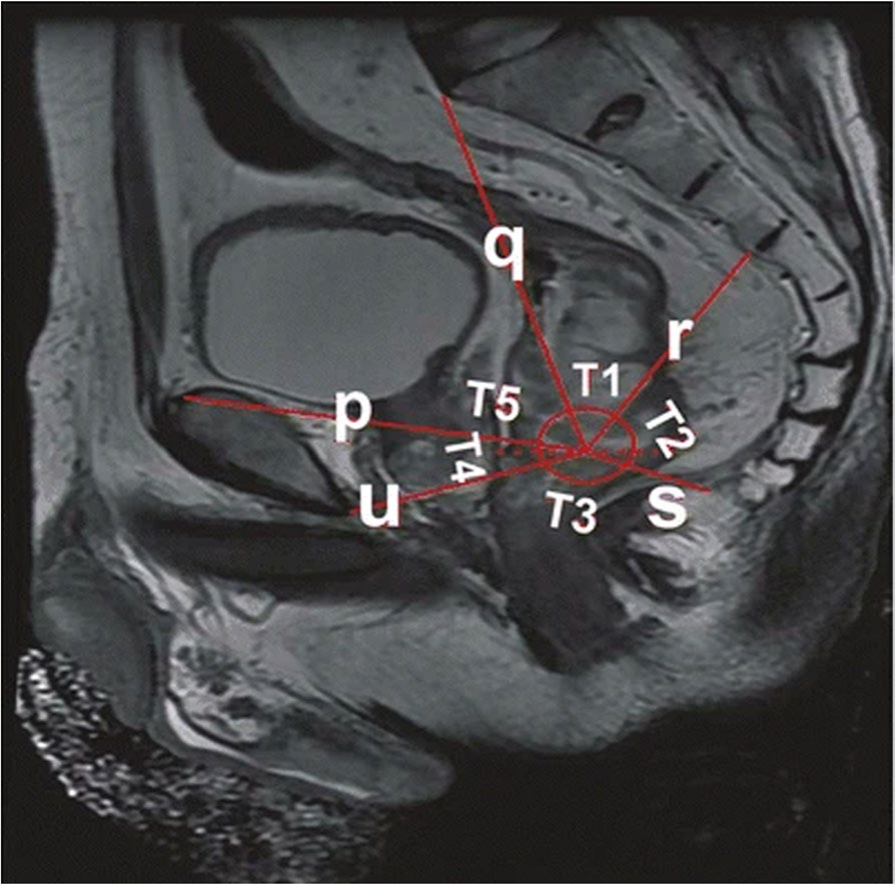
Sagittal magnetic resonance image of the pelvis: **l** T4 angle, i.e., the angle between the upper and lower borders of the pubic symphysis with the lower border of the tumor as the vertex [31]

## Influence of a difficult pelvis on rectal cancer surgery

Laparoscopic TME (lapTME), based on open TME (opTME), has become a relatively mature surgical procedure after decades of development [32]. It has the advantages of minimal invasiveness, such as the capacity for causing less abdominal wall trauma, less postoperative pain, and a better aesthetic appearance of the abdominal wall, and it is currently one of the main ways to treat rectal cancer [33, 34]. The laparoscopic lens can also be extended into the pelvis, facilitating the availability of a multi-angle surgical field that cannot be achieved with open surgery. However, laparoscopic rectal cancer surgery is significantly more difficult to perform than other surgical techniques. On the one hand, the anatomical position of the rectum is located in the deep part of a narrow funnel-shaped pelvis, the surrounding tissues are complicated, and the space for the operator to operate is limited. On the other hand, laparoscopic surgery requires complex procedures such as incision, dissociation, hemostasis, and anastomosis to be performed through inflexible long-handled endoscopic manipulation instruments, which is completely different from traditional manual operations and lacks tactility. Previous randomized controlled trials have shown that laparoscopic surgery may lead to adverse outcomes, such as low-quality TME specimens or an increased rate of positive circumferential margins [35, 36]. Another recent meta-analysis also concluded that there is a significantly greater risk of unsuccessful laparoscopic surgery compared to open surgery [37]. It can be seen that the influence of the pelvic space on laparoscopic rectal cancer surgery cannot be ignored.

### Operation time

Operation time is the most convenient measure of the surgical difficulty. A multivariate analysis by Zhou et al. showed that body mass index (BMI), tumor height, lymph node metastasis, anteroposterior diameter of the pelvic entrance, anteroposterior diameter of the pelvic outlet, pubic symphysis height, sacrococcygeal curvature depth, sacrococcygeal–pubic angle, and suprapubic border to the distance from the tip of the coccyx are the main factors influencing the operation time [19]. In addition to relevant clinicopathological parameters, a wider, shallower, and less curved pelvis may reduce the operation time and amount of intraoperative blood loss. In contrast, the surgical difficulty may be increased when treating patients with deeper, narrower, or larger sacrococcygeal pelvises [29]. Yang et al. also believed that narrow pelvic parameters would affect the difficulty of surgery for middle and low rectal cancer. Yang’s prediction model pointed out that a larger T4 angle (taking the lower edge of the tumor as the vertex, with the angle existing between the upper and lower edges of the pubic symphysis) and a greater distance between the tumor and the anal verge support a shorter operation time and suggest the patient is a better candidate for anus-preserving surgery [31]. Other studies predicting the difficulty of laparoscopic rectal cancer surgery still concluded that a smaller pelvis increased the operative time [5, 8]. Based on this, several recent studies have established models for predicting surgical difficulty, but external validation is still lacking [26–28]. In addition, it is worth mentioning that, anatomically, women have wider and shallower pelvises than men, so such surgical procedures may be easier to perform in female patients, and some research has confirmed this point [7]. Atasoy et al. also concluded that male sex, a deeper pelvis, and a smaller abdominal cavity all lead to longer operative times [7]. However, the broader literature offers conflicting views. The study by Zhang et al. found that sex is not one of the factors affecting the difficulty of surgery. Although the “deep and narrow pelvis” seen in men increases the difficulty of surgery to a certain extent, the presence of uterine appendages uniquely in women also affects the surgical field of vision and increases the operation time [22]. To sum up, it seems that the degree of difficulty of surgery cannot be judged solely by sex, and pelvic measurements should be accurately performed to conclude the true level of complexity that may be expected.

At present, the impact of a difficult pelvis on operation time is also controversial. An earlier study reported there is no association between pelvic size and operative time [38], suggesting that a “narrow pelvis” is not a contraindication to laparoscopic rectal surgery. Ogiso et al. studied patients undergoing laparoscopic rectal cancer resection, using 3D volume–rendering images to measure the relevant pelvic dimensions (i.e., the anteroposterior and transverse diameters of the pelvic entrance, anteroposterior and transverse diameters of the pelvic outlet, and pelvic depth), and their results showed that there was no correlation between these five parameters and operation time. At the same time, their study also confirmed that sex was not a factor that significantly affected surgical outcomes.

### Surgical quality

The quality of TME is one of the most important prognostic factors for local recurrence of rectal cancer. In a recent study with a large sample size [16], an analysis of 198 patients undergoing surgery for mid–low rectal cancer found that the angle A5 (which is the angle between the line connecting the superior and inferior borders of the pubic symphysis and the line connecting the midpoint of the superior border of the pubic symphysis to the sacral promontory) has a significant effect on the integrity of the TME specimen. Ferko et al. believed that the sharper the A5 angle, the more difficult the operation and the worse the TME specimen quality would be. In addition, Zur Hausen et al. determined that a shorter interspinous diameter of the ischial spine increased the probability of TME specimen damage. The ischial spine spacing shows a trend toward an increased risk of TME specimen quality decline, and a shorter ischial spine spacing may be an independent risk factor for poor surgical quality [24]. Other research has also confirmed this [26]. Compared to open surgery, in the most distal anatomy of the mesorectum, lapTME is difficult to perform due to the curved angle of the sacrococcygeal bone, which can easily lead to low-quality TME specimens and increased rates of circumferential resection margin positivity [24, 30]. An international multicenter, randomized controlled trial comparing taTME and lapTME also showed that lapTME makes it more difficult to achieve complete TME specimens with clear circumferential margins in low-to-medium rectal cancer [39].

In addition to TME specimen quality, circumferential resection margin is also one of the salient parameters to evaluate the quality of the surgery. However, several recent studies have found that the usability of Pelvic parameters in predicting circumferential resection margin involvement remains debatable [40–42]. Yamaoka et al. investigated the factors affecting CRM status and the importance of computed tomography (CT) pelvimetry in predicting CRM involvement in laparoscopic resection of middle and lower rectal cancer. The results showed that the effect of pelvic anatomic parameters on CRM involvement was not found to be significant [40]. It was found that tumor height from the anal verge (*p* = 0.004), tumor size (*p* < 0.001), and gender (*p* = 0.033) were significant risk factors for CRM involvement. Another study found that pelvic dimensions on preoperative imaging can identify poor-quality resections after laparoscopic low anterior resection for mid- and low rectal cancer, but only a small fraction involved CRM [41]. It appears that the CRM involvement is more influenced by the tumor itself than by the pelvic parameters [43].

## Complications

The study by Zhou et al. found that, in an open rectal cancer operation, the anteroposterior diameter of the middle pelvis, the anteroposterior diameter of the pelvic outlet, the ischial spine diameter, and the pelvic depth correlated with the amount of intraoperative blood loss. In contrast, the pelvic parameters seem more meaningful to the Miles procedure, which suggests that the deeper the pelvis is, the more difficult it is to operate on [19]. Other studies have also linked a narrow pelvis to an increased incidence of anastomotic leakage [6, 7]. In their study, Atasoy et al. analyzed the pelvic depth as an independent predictor of anastomotic leakage [7]. Similar to the previous investigation, Yamaoka et al. found that a larger mesorectal fat area was significantly associated with the positivity rate of anastomotic leakage in rectal cancer surgery and the mesorectal fat area could be used as a predictor of the technical difficulty of TME [23]. On this basis, Tsuruta et al. created a completely new index, known as the pelvic index, which was defined as the ratio of the difference in distance between the ischial spines and the diameter of the mesorectum to the pelvic depth at the level of the seminal vesicles [25]. Through experimentation, these authors found that the pelvic index of the anastomotic leakage–positive group was significantly lower than that of the anastomotic leakage–negative group. Comparisons between these two groups at the pelvic index cutoff boundaries showed significant differences. The greater the difference in the distance between the ischial spines and the diameter of the mesangial fat and the smaller the pelvic depth, the easier it was to perform surgical manipulation in the pelvis.

Unlike in the previous study, Zur Hausen et al. did not observe an association between pelvic parameters and anastomotic leakage or urinary tract dysfunction, which suggested that anastomotic leakage was associated with anemia [24]. Their study of 74 patients undergoing low anterior resection, while confirming a significant correlation between ischial spine spacing and TME quality, also found that anastomotic leakage appears to be more affected by clinical factors such as anemia, not by pelvic size.

## Discussion

In summary, an anthropoid pelvis with large vertical depth, short transverse longitude of the small pelvis and large curvature of sacrococcygeal bone is a difficult pelvis. However, the author argue that previous studies of pelvis difficulty have focus on the bone structure itself, and that the operable space of the pelvis during rectal cancer surgery is also affected by the pelvic adipose tissue. Therefore, all the pelvis with limited operable space in rectal cancer surgery is a difficult pelvis.

Generally speaking, obesity reduces the relative space in the abdominal cavity, making the operation more difficult to perform, and several studies have confirmed a negative effect of BMI on rectal surgery [19, 26, 38, 44]. However, Chen et al. found through multivariate regression analysis that the larger the proportion of the mesorectal fat area, the lower the probability of complete surgical resection of the mesorectum would be, and the effect of BMI on the outcome of rectal surgery was not significant [45]. The mesorectal fat area appears to be a better predictor than BMI of surgical quality and the difficulty of laparoscopic rectal surgery in obese patients. Similarly, Levic et al. found that BMI was not a significant predictor of poor outcome in patients undergoing laparoscopic rectal cancer surgery, although patients with BMIs of ≥ 30 kg/m^2^ had a concomitant increase in intraoperative blood loss. The explanation for this is that the distribution of the visceral fat area is individualized in different patients [44]. BMI reflects the overall degree of obesity, while the fat content in the abdominal cavity, especially around the rectum, has a major impact on the difficulty of surgery. As such, mesorectal fat area fraction may be a better indicator of the difficulty of operation. Although some previous studies also took into account mesorectal fat area, it was only expressed in a one-dimensional scale due to the measurement conditions [45]. The authors suggest that the measurement method could be improved to use the difference between the pelvic cross-sectional area at the level of the ischial spine and mesorectal fat area as the main prediction parameter. This is a lot more straightforward than BMI. Furthermore, most of the previous studies measured the relevant parameters on pelvic imaging screenshots. 

Furthermore, complex relationships might need more features to build a proper prediction model. The surgical difficulties in rectal cancer cannot be predicted using a single parameter with high accuracy. Therefore, the composite approach, such as nomogram, machine learning (ML), and artificial intelligence (AI), may be needed to overcome the problem [46]. While most of the previous related studies also focused on multiple parameters, they did not form a scientific composite index to assess the difficulty of surgery. The use of different difficulty measures also limits comparisons between studies [13]. Four criteria of a recent study were selected for assessment: operative time, intraoperative blood loss, postoperative hospital stay, and postoperative complications. Using multivariate analysis, they found the factors significantly associated with surgical difficulty were BMI, pelvic inlet and intertuberous distance, and a nomogram model was established with the selected parameters for predicting the probability of high surgical difficulty. This objective method would provide a visualization tool to effectively predict the probability of surgical difficulty in RC [21]. Similarly, the study of T. Yamamoto et al. also used multiple criteria to comprehensively define the difficulty of surgery [47]. Multivariable analysis indicated that surgical difficulty was associated with BMI, tumor size, anorectal angle, and pelvic outlet. All of these features were used to devise a four-variable scoring model to predict surgical difficulty. On the other hand, to define the best treatment option and optimize patient outcome, there is a growing interest in artificial intelligence applications in medicine, and imaging is by no means an exception [48]. In this setting, a post processing quantitative technique (Radiomics), which has been frequently and successfully coupled with artificial intelligence and machine learning, appears particularly promising [49, 50]. So far, however, few studies have been carried out on predicting the difficulty of laparoscopic rectal cancer surgery using these new methods. There is still a long road ahead. 

### Outlook

Rectal cancer is a disease that seriously endangers human beings, and surgery is still its main treatment method. The use of lapTME for treating middle and low rectal cancer still carries some challenges when performed in the difficult pelvis [51, 52]. When encountering patients with mid–low rectal cancer and a narrow pelvis, exposure to the surgical field, separation of deep tissue, and rectal anastomosis are extremely difficult.

With the continuous development of minimally invasive technology in recent years, taTME and robotic rectal cancer surgery have gradually matured. taTME combines the bottom-up surgical strategy with the concept of TME, which may be easier to use to complete low rectal dissection and mesentery resection in the deep and narrow pelvis, and its core advantage is ensuring the quality of the resection of the distal mesentery of the rectum. At the same time, since the surgical approach starts from the pelvic floor, taTME can better ensure that the organs and nerves of the pelvic floor are not damaged, and this surgical method involves no auxiliary incision in the abdominal wall, in line with the concept of minimal invasiveness. In some studies, taTME has also yielded better short-term clinical outcomes, such as a lower incidence of anastomotic leakage, wider circumferential margins, and greater TME specimen integrity [8, 9]. However, there is still a lack of large-scale studies on long-term prognosis, and its application value remains to be verified [53]. In addition, the robot has multiple 360° rotatable mechanical arms, which can perform operation steps that cannot be completed by human hands and conventional laparoscopic instruments [16], thereby overcoming the difficulties caused by pelvic anatomy. At the same time, the robot offers a clearer and magnified 3D image than laparoscopy, which makes it easier to distinguish the anatomical structures during the operation and reduces the possibility of damage to the pelvic nerves, blood vessels, ureters, and other structures. The robot’s multiple arms also reduce the operator’s dependence on assistants and the difficulty of learning in low rectal cancer surgery. Several studies have found that a narrow pelvis does not lead to a significant increase in operative time [51]. This suggests that a robotic system could provide surgeons with greater comfort while performing surgery on a difficult pelvis and potentially overcome challenges associated with difficult pelvic anatomy. The short-term outcomes of robotic rectal cancer surgery appear to be superior to those of lapTME [54]. However, these robots are expensive, and, at present, considering the additional economic and time overhead, robotic surgery may be selectively applied only to those patients who might most benefit from this new technology.

With the continuous advancement of medical imaging, the accuracy of preoperative assessment of the pelvic shape has increased, and imaging technology has high clinical value in rectal cancer–related applications. Surgeons often rely on the visual aid of medical imaging techniques, such as MRI or CT, to plan surgical procedures. However, due to the anatomical complexity of the surgical site, differences between the real anatomy and virtual images still exist, and the success of surgical procedures is largely dependent upon the surgeon’s previous training and experience. A 3D printed model of the patient’s anatomy enables personalized preoperative planning for such scenarios. At present, some institutions have applied 3D printing models to surgical planning and training [55–57]. It is believed that, in the future, preoperative imaging assessment will not only be used for accurate pelvic measurement but also widely for planning purposes, such as simulating rectal cancer surgery, to improve surgical outcomes and reduce medical errors.

In summary, there are many anatomical parameters of the pelvis, and there are many surgical methods available for rectal cancer. The methods for evaluating the difficulty of rectal cancer surgery are different, and the conclusions of those methods are still controversial. The risk of adverse outcomes associated with the unwarranted and uncontrolled use of these new technologies should be avoided in the clinic so that patients can receive the best and most beneficial treatment for them. Therefore, the surgeon should evaluate the difficulty of the operation in combination with the findings of the preoperative examination, then choose the appropriate surgical method for individual cases, such as lapTME, robotic surgery, or taTME, or even pass the case on to a more experienced surgeon.

## Data Availability

Not applicable.
